# A patient-centered qualitative evaluation of meaningful change on the NSAA and PUL in Duchenne Muscular Dystrophy

**DOI:** 10.3389/fneur.2025.1509174

**Published:** 2025-03-04

**Authors:** Aviva Gillman, Teofil Ciobanu, Louise Barrett, Evan W. Davies, Alexander P. Murphy, Alex Johnson, Jessica Mills, Phoebe Heinrich, Krystian Przydzial, Bethany Ewens, Gerrit Vandenberg, Stefan Cano, Anna Mayhew

**Affiliations:** ^1^Modus Outcomes, a THREAD Company, London, United Kingdom; ^2^F. Hoffmann-La Roche, Basel, Switzerland; ^3^Roche Products Ltd., Welwyn Garden City, United Kingdom; ^4^Duchenne UK, London, United Kingdom; ^5^John Walton Muscular Dystrophy Research Centre, Translational and Clinical Research Institute, Newcastle University and Newcastle Hospitals NHS Foundation Trusts, Newcastle upon Tyne, United Kingdom

**Keywords:** Duchenne Muscular Dystrophy, patient-reported outcomes, qualitative research, rare disease, meaningful change

## Abstract

**Background:**

Duchenne Muscular Dystrophy (DMD) is a rare X-linked genetic disorder caused by mutations in the dystrophin gene. The North Star Ambulatory Assessment (NSAA) and Performance of Upper Limb (PUL) are used to measure motor performance in ambulatory and non-ambulatory individuals, respectively. There is limited published qualitative evidence on what constitutes a meaningful change on either instrument. The aim of this study is to understand meaningful change in functional abilities as measured by the NSAA and PUL at different ability levels from the perspective of individuals with DMD, caregivers of individuals with DMD, and clinicians.

**Methods:**

The study utilized a non-interventional, descriptive, cross-sectional qualitative design consisting of 69 semi-structured interviews, including individuals with DMD (*n* = 18), caregivers of individuals with DMD (*n* = 51), and neuromuscular physiotherapists (*n* = 2) to understand meaningful change on the NSAA and PUL.

**Results:**

The results for both instruments indicated that: (i) items that are meaningful differ based on ability level; (ii) maintaining function in lower and upper limbs is the ultimate goal; (iii) meaningful change is often reported in relation to gain or loss of specific function, as opposed to number of total points on the scale; and (iv) losing one scale point corresponds to either a partial or full loss in function and activity, which has differing impacts on daily life.

**Conclusion:**

The perception of meaningful change in DMD as measured by the NSAA is influenced by ability levels and ambulatory function, with participants describing their need to maximize certain abilities, maintain function, and retain independence. For the PUL, participants underscored the importance of maintenance of their functional abilities, and highlighted key themes related to maintaining independence in ADLs, reaching, eating/drinking, and finger function for technology use across score categories.

## Introduction

Duchenne Muscular Dystrophy (DMD) is a rare X-linked genetic disorder caused by a mutation in the dystrophin gene (1) that leads to severe progressive muscle weakness and premature death. Muscle weakness affects proximal limb muscles before progressing distally, with symptoms evident in lower extremities before impacting upper extremities (2–4). Individuals with DMD first have difficulty with activities such as running, jumping, and getting up off the floor, and diagnosis usually follows at around four and a half years of age (5). Individuals with DMD typically lose ambulatory function due to muscle weakness between the ages of 12 and 14 (3). Following this, their upper limb functions are affected – although weakness can also occur prior to loss of ambulation (LOA) – and certain comorbidities may develop over the course of decades such as respiratory issues and cardiomyopathy (3). Individuals with DMD have a reduced life span of 28 years on average (6).

The current standard of pharmacologic treatment for DMD includes corticosteroids to delay LOA and other secondary complications (2, 7, 8). Corticosteroids, however, have a multitude of side-effects associated with long-term use that impact quality of life (8). Novel disease-modifying therapies, such as gene transfer therapies, offer the potential to ameliorate the disease by targeting the underlying cause and producing a functional form of dystrophin protein (2).

Clinical outcome assessments (COAs) are regarded as crucial to evaluating the benefit of any new potential therapeutic treatment as well as in clinical practice to demonstrate or predict disease progression. Two clinician-reported outcome assessments (ClinROs) are commonly used to measure physical function in DMD: the North Star Ambulatory Assessment (NSAA) for ambulatory individuals (9) and the Performance of Upper Limb Version 2.0 (PUL) for weaker ambulatory (based on entry item less than six) and non-ambulatory individuals (10).

In order to demonstrate clinical benefits, regulatory bodies require evidence that the change observed in patient physical functioning is clinically meaningful. Meaningful change can broadly be defined as “the perceived meaning of health outcome score changes or differences based on the target population’s perception” (11, 12). Establishing clinically meaningful change on COAs is commonly done using anchor-based and distribution based-quantitative analysis. Recent regulatory guidance on the issue of meaningful clinical benefit highlights the importance of also obtaining patient input (e.g., eliciting patient definitions of symptom improvement, stability, or worsening) when establishing meaningful outcomes or change on COAs (13, 14). Understanding the patient, clinician and caregiver perspective offers valuable insight into what is considered an important amount of change, why it is important, and how it translates into daily functioning (11). However, establishing meaningful change from a qualitative perspective is a relatively new methodology and has associated challenges, such as the unique individual differences of the lived experience of each patient, and their own perception of what is considered meaningful.

To date, there is limited published evidence on quantitative estimates of meaningful change on the NSAA (15, 16) and no published estimates of meaningful change on the PUL (10). Furthermore, no qualitative data on meaningful change has been published for either instrument. This study enhances the existing evidence base on conducting meaningful change analysis by offering the first qualitative perspectives of change on the NSAA and PUL.

Specifically, the research intended to understand what constitutes meaningful change in functional abilities as measured by the NSAA and PUL at different ability levels from the perspective of individuals with DMD, caregivers of individuals with DMD, and clinicians. Furthermore, the study sought to provide evidence of how individuals experience symptoms and change in symptoms over time, with a focus on progression of disease as measured by the NSAA and PUL on an item and total score level.

## Materials and methods

This research adheres to the COREQ-checklist for reporting qualitative research (see Supplementary Table 1) (17).

### Sample and recruitment

The study utilized a non-interventional, descriptive, cross-sectional qualitative design consisting of 69 semi-structured interviews with individuals with DMD and their caregivers, as well as two interviews with neuromuscular expert physiotherapists.

A convenience sample was recruited by Patient Advocacy Groups (PAGs) in the US, UK, Canada, and Australia. Recruitment letters were posted on PAG websites and circulated using email and social media. Potential participants were deemed eligible if they met the following criteria (for individuals with DMD): diagnosis of DMD, aged 13 or older (if between 13 and 18, a guardian had to be present), male, knew their most recent NSAA/PUL score (or could provide it prior to the interview), able to speak, read, and write English, able and willing to participate in a 1-h interview, and willing to have their interview audio and/or video recorded. Potential participants were excluded if they met any of the following criteria: they were female, possessed insufficient knowledge of English, or were enrolled in a current therapeutic clinical trial that restricted access to NSAA / PUL scores.

The following criteria were used to determine the eligibility of caregivers as potential participants: the individual was aged 18 years or older, the primary caregiver of an individual aged 4 or older with a diagnosis of DMD, knew the most recent NSAA/PUL score of the individual they care for (or could provide it prior to the interview), able to speak, read, and write English, able and willing to participate in a 1-h interview, and willing to have their interview audio and/or video recorded. Potential caregiver participants were excluded if they met any of the following criteria: the individual they care for with DMD was female, they possessed insufficient knowledge of English, or the individual they care for was enrolled in a current therapeutic clinical trial that restricted access to NSAA/PUL scores.

The study protocol and documents were approved with continuing oversight by the Western Institutional Review Board (WIRB)-Copernicus Group (WCG IRB) for the US and Canada and Bellberry Limited in Australia. Exemption from NHS Research Ethics Committee (REC) approval was obtained in the UK. All participants provided informed consent.

### Clinical and expert input

One clinical expert consultant (AMa) provided their expertise throughout the project by reviewing study materials and providing feedback and input on results and recommendations. Representatives from PAGs also provided input on all study materials. Two further physiotherapists were involved as interview participants.

### Overview of instruments

The NSAA is a 17-item ClinRO used to measure lower-limb function in ambulant children with DMD (9); it is scored on a scale of 0–2: a score of 0 indicates the individual is unable to complete the item, 1 indicates they can do it with modification, and 2 indicates they can complete the item fully. The PUL is a 22-item ClinRO that provides an upper extremity score for individuals across a range of abilities from ambulatory males who may exhibit early signs of upper limb weakness to older weaker adults with limited arm function (10). The PUL has an entry item to identify the starting point based on current upper limb ability (not included in total score), and three subscales: high-level shoulder dimension, mid-level elbow dimension, and distal wrist and hand dimension. The scoring is the same as in the NSAA, apart from two items (15 and 22), which have only two response options: 0 = unable or 1 = complete fully.

### Interview procedures

Individuals were assigned to a NSAA or PUL interview depending on which instrument they were most recently measured on. Four participants provided both scores at screening and were assigned to an interview group in consultation with AMa to ensure they were placed in the appropriate group based on their ability as well as ensuring perspectives were gained across a wide range of score levels. Interviews were semi-structured using a 1:1 interviewing approach. Interviews were conducted online by researchers experienced in qualitative methods. They lasted approximately 60 min, were audio recorded and transcribed verbatim, and entered into ATLAS.ti software version 23 (ATLAS.ti Scientific Software Development GmbH). A patient-friendly modified version of each instrument was developed in collaboration with one of the instruments’ developers and shared with participants prior to their interview to gain an approximate understanding of their current functional ability on each item. These patient-reported scores were used only to help guide the interview, by avoiding starting with items participants could no longer complete, and were collected in addition to the clinician-reported scores provided at screening. The modified instruments were not used as a substitute for clinician-reported scores. These versions had the exact same number of items and response options as the original versions, however with the addition of pictures illustrating the tasks, and the revision of definitions of each scoring category for clarity and simplification (e.g., score 1 = “I can do this but with some changes to how I do it e.g., with difficulty”).

In the semi-structured interviews, researchers sought to gain an understanding of meaningful change at an item-level on both the NSAA and the PUL. For each instrument, participants were asked about which items they regarded as most and least important; which items they would most want to improve or maintain; whether their ability on each item had improved, declined, or been maintained over the past year (and whether that was important); the impact of any recent decline or improvement; and the impact of any potential future decline, improvement, or maintenance (and whether that would be important). The interview guide was tested internally prior to the interviews and minor revisions were made following the first few interviews to improve the acceptability of the guide.

### Qualitative data analysis

Researchers conducted inductive line-by-line coding of de-identified transcripts using ATLAS.ti software and analyzed the data thematically (18). A coding guide was used to ensure consistency of approach between a team of three coders per instrument. Following independent parallel coding for the first two interviews, the team met several times to discuss the coding process and any inconsistencies, updating the coding guide as needed.

All participants were asked about recent or future change on items in general, however, a change in score related to points was investigated when raised organically. For this reason, frequencies were not always available, and these results were interpreted descriptively. When participants discussed a change in score, this was classified as either:

- Partial loss (worsened function): item score decreases from 2 to 1
- Full loss (worsened function): item score decreases from 1 to 0 or 2 to 0
- Improvement (improved function): item score increases from 0 to 1 or 1 to 2
- No change: item score stays the same: 0 to 0 or 1 to 1 or 2 to 2

The results were grouped by score into three ability levels per instrument to understand key themes of meaningful change across disease progression.

- NSAA: high (25–34); mid (15–24); low (0–14)
- PUL: high (32–42); mid (21–31); low (0–20)

The categories were devised in collaboration with the expert consultant, prior to any analyses, and were based on the disease trajectory of DMD and clinical expertise of disease progression. These groups should not be relied upon as clinical indicators of ability without further investigation and were simply used to examine patterns in the data. Each quotation is identified by the transcript identification number, country, and whether they were a caregiver or individual with DMD.

## Results

### Sample

A total of 71 individuals participated in this study, including two neuromuscular expert physiotherapists, 18 individuals with DMD, and 51 caregivers of individuals with DMD. One physiotherapist was from the UK and one from the US, and they had a combined 30+ years in practice.

In the NSAA sample (*n* = 35), there were four individuals with DMD and 31 caregivers. Two dyads (caregiver and son) were interviewed separately, therefore, 35 interviews represented 33 unique individuals with DMD. The individuals in the caregiver-reported interviews (*n* = 33) had an average age of 10 (range 4–17), and the individuals with DMD (*n* = 4) who participated themselves had an average age of 14 (range 13–16). In the PUL sample (*n* = 34) there were 14 individuals with DMD and 20 caregivers, including nine dyads, therefore 34 interviews represented 25 individuals with DMD. For the PUL, the individuals in the caregiver-reported interviews had an average age of 16.4 (range 10–28); the 14 individuals who participated in interviews themselves had an average age of 16.9 (range 13–28).

Full descriptions of individuals with DMD and caregivers can be found in Tables 1, 2. A breakdown of the age, score, and ability level for both NSAA and PUL can be found in Table 3. Data on non-participation is available in Supplementary Table 2.

**Table 1:** Sample characteristics of individuals with DMD across NSAA and PUL.

Individuals with DMD	NSAA		PUL	
	All individuals with DMD *n* = 33	Patient interviews *n* = 4	All individuals with DMD *n* = 25	Patient interviews *n* = 14
Age in years				
Mean, SD	10 (3.4)	14 (1.2)	16.2 (3.9)	16.9 (3.9)
Range	4–17	13–16	10–28	13–28
Age at diagnosis				
Mean, SD	3.5 (2.4)	4.8 (0.8)	5.1 (3.2)	4.5 (2.4)
Range	0–10	4–6	1–15	1–9
Years taking corticosteroids				
Mean, SD	5.0 (3.3)	6.0 (4.2)	10.5 (3.7)	11.5 (4.0)
Range	0.5–12	2–12	5–21	6–21
Living situation, n (%)				
Live with parents	30 (91)	4 (100)	25 (100)	14 (100)
Living with partner spouse, family, or friend	1 (3)	–	–	–
Social housing	1 (3)	–	–	–
Foster care	1 (3)	–	–	–
Education, n (%)				
Student, full time	24 (73)	4 (100)	18 (72)	11 (79)
Student, part time	1 (3)	–	1 (4)	1 (7)
Unemployed	–	–	2 (8)	2 (14)
Missing	8 (24)	–	4 (16)	0 (0)
Level of education, n (%)				
Elementary/primary school	21 (64)	1 (25)	3 (12)	2 (14)
Secondary/high school	7 (21)	2 (100)	13 (52)	9 (64)
Middle school			2 (8)	1 (7)
Some years of college/university of A–levels	–	–	3 (12)	2 (14)
College or university degree	–	–	1 (4)	–
Other	2 (6)	1 (25)	0 (0)	–
Missing	3 (9)	–	3 (16)	0 (0)
Ambulatory status, n (%)				
Able to walk without aid	26 (79)	2 (50)	2 (20)	–
Able to walk with aid	6 (18)	2 (50)	3 (12)	2 (14)
Unable to walk with or without aid	–	–	19 (76)	12 (86)
Missing	1 (3)	–	–	–
Corticosteroid-treated, n (%)				
Yes	31 (94)	4 (100)	24 (96)	14 (100)
No	2 (6)	–	1 (4)	–
Missing			0 (4)	0 (0)
Corticosteroid regime, n (%)				
Daily	28 (85)	4 (100)	20 (80)	10 (79)
10 days on, 10 days off			3 (12)	2 (14)
2 days a week	3 (9)	–	2 (8)	1 (7)
Missing	2 (6)	–	–	–

‘All individuals with DMD’ refers to both the subjects of caregiver interviews and individuals themselves who were interviewed. Patient interviews refer specifically to individuals with DMD who were interviewed. SD, Standard Deviation.

**Table 2:** Sample characteristics of caregivers across NSAA and PUL.

Caregivers	NSAA, *N* = 31	PUL, *N* = 20
Age/years	Mean (SD)	Mean (SD)
Mean, SD	42.87 (7.09)	46.7 (6.23)
Range	28–56	34–56
Sex, n (%)		
Female	24 (77)	17 (85)
Male	7 (23)	3 (15)
Relationship to individual with DMD, n (%)		
Mother	22 (71)	17 (85)
Father	7 (23)	2 (10)
Grandparent	1 (3)	1 (5)
Foster parent	1 (3)	–
Ethnicity, n (%)		
White	24 (77)	17 (85)
Hispanic or Latino	3 (11)	1 (5)
Black/African/Caribbean	1 (3)	–
Native Hawaiian or other pacific Islander	1 (3)	–
Asian	1 (3)	4 (20)
Mixed or multiple ethnic groups	–	2 (10)
Prefer not to answer	1 (3)	–
Employment status, n (%)		
Employed, full-time	14 (45)	7 (35)
Employed, part time	5 (17)	6 (30)
Volunteer, part-time	2 (6)	–
Student, full time	1 (3)	–
Full time carer	8 (26)	7 (35)
Unemployed	1 (3)	–
Caregiver level of education, n (%)		
College or university degree	11 (36)	14 (70)
Postgraduate degree	11 (36)	2 (10)
Secondary/high school	3 (11)	2 (10)
Some years of college/university	3 (11)	1 (5)
Some years of graduate degree (master’s or PhD)	1 (3)	–
Technical or vocational degree	1 (3)	1 (5)
Other: GED	1 (3)	–

SD, Standard Deviation; GED, General Educational Development.

**Table 3:** NSAA and PUL scores and age by ability level.

		Clinician-reported score			Patient-reported scores		
Ability level	Total sample size	Age	Score	Sample size	Age	Score	Sample size
NSAA	n (%)	Mean (SD)		n (%)	Mean (SD)		n (%)
High (25–34)	13 (37)	8.9 (2.6)	30.2 (2.0)	12 (36)	8.6 (2.4)	29.1 (4.8)	12 (36)
Mid (15–24)	11 (31)	10.0 (4.1)	19.1 (3.7)	11 (33)	10.2 (3.7)	18.5 (2.7)	13 (39)
Low (0–14)	11 (31)	11.5 (3.1)	10.4 (2.6)	10 (30)	12.0 (3.5)	8.8 (4.8)	8 (24)
PUL							
High (32–42)	14 (41)	14.4 (2.1)	36.4 (3.8)	9 (36)	14.6 (1.8)	36.1 (4.0)	11 (44)
Mid (21–31)	11 (32)	15.1 (3.6)	25.2 (3.1)	9 (36)	15.8 (3.8)	26.5 (3.7)	8 (32)
Low (0–20)	9 (26)	20.0 (4.0)	15.1 (4.4)	7 (28)	19.8 (5.2)	15.0 (4.7)	6 (24)

Breakdown of sample based on ability level scores for both the NSAA and PUL. Based on number of unique individuals with DMD; *n* = 33 NSAA, *n* = 25 PUL. SD, Standard Deviation.

### Clinical definition of meaningful change on the NSAA and PUL

The findings indicated that meaningful change thresholds may be different for individuals based on the way each instrument operates. One of the clinicians defined meaningful change as “a change in function that has a significant impact on a patient’s everyday life, but that can be different per patient” (CL-1). Furthermore, items will be weighted differently in terms of importance at different stages of the disease, and that, “looking at the items that change would be more important than the total score” (CL-2). For example, clinicians noted that some of the items in the PUL required greater differentiation to mark degrees of impairment. According to one clinician, “someone who can reach over their head versus someone who cannot get their hand to their mouth? […] The less functional you are, the more these points matter” (CL-2).” Clinicians also expressed concern that the scale may not represent equal importance to participants; a full loss on an individual item, for example, might be more meaningful than a partial loss on both instruments: “I think most patients do not notice that until it’s significant enough, where they are about to lose it, or their compensations are so great that it’s fatiguing to do it” (CL-2).

### NSAA results

A key theme emerged for each ability category in the NSAA data (reported directly below) as well as three key findings related to meaningful change: (1) the concept of meaningful change varies depending on functional ability; (2) maintenance of function is important for all, but key items shift across ability levels; and (3) partial and full loss in items are associated with different impacts.

#### Key themes by ability scores

**Figure 1:**
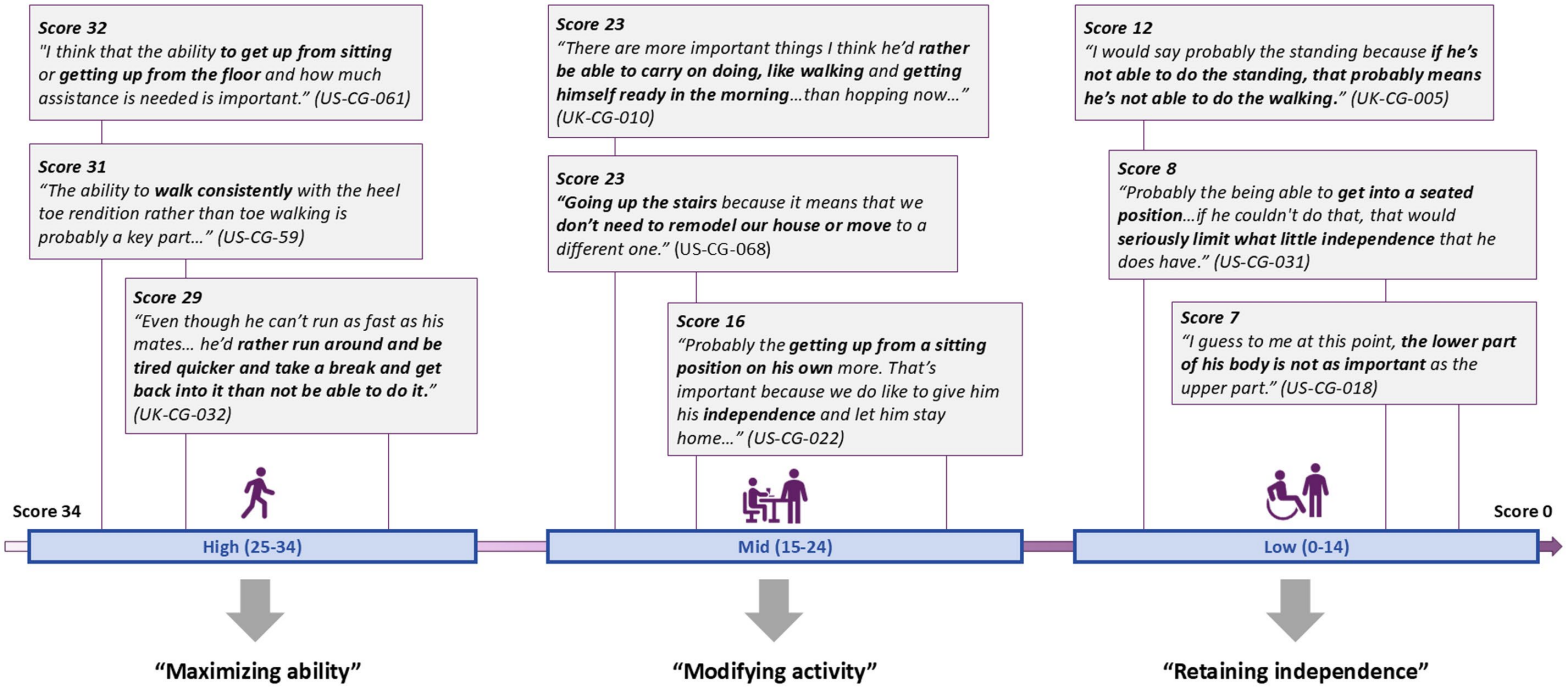
NSAA ability scores and associated themes.

Figure 1 displays themes and quotes related to the meaningfulness of NSAA items across disease progression. Individuals with high NSAA scores emphasized the importance of ‘maximizing abilities’ in relation to keeping up with peers, socializing, and being active. Speed was important for items such as running or getting up off the ground quickly: *“he does not want to be held back and he does have siblings as well and so, he wants to be just as quick as they are because they are all close in age” (US-CG-061).* Individuals with mid NSAA scores spoke about the importance of ‘modifying activities’ and focused on limiting activity to preserve function in items which had declined in the past year, such as walking, running, and going up and down stairs. A caregiver noted “*he does fatigue a lot sooner so then that’s the thing we have to watch for him is not to let him walk too much because then his legs will get tired, and he’ll have a chance of a fall” (US-CG-022)*. In the low score group, ‘retaining independence’ was important, particularly in completing activities of daily living (ADLs). One caregiver mentioned getting into a seated position independently would be most important, “*because if he could not do that, that would seriously limit what little independence that he does have…” (US-CG-031).*

#### Meaningful change and functional ability

Walking was identified as the most important item by almost all participants across the spectrum of ambulatory ability, but the significance of other items was contingent upon the varying functional ability of ambulatory individuals in each ability group (Table 4). The data showed that, for example, getting off the floor may be more meaningful to someone going through a transitional phase compared to an individual who is still walking and running, in which case jumping may be more meaningful. Quotes from caregivers about individuals at different ability levels exemplify this:

**Table 4:** Most important items: frequencies for participants across NSAA ability score categories high, mid, and low.

Item	High (25–34) (*n* = 13)	Mid (15–24) (*n* = 11)	Low (0–14) (*n* = 11)
01 Stand	2 (15%)	5 (45%)	4 (36%)
02 Walk	8 (62%)	7 (64%)	7 (64%)
03 Stand up from chair	5 (38%)	2 (18%)	2 (18%)
04/05 Stand on one leg	–	1 (9%)	–
06/08 Climb box step	3 (23%)	7 (64%)	2 (18%)
07/09 Descend box step	2 (15%)	5 (45%)	3 (27%)
10 Lifts head	3 (23%)	–	1 (9%)
11 Gets to sitting	3 (23%)	4 (36%)	4 (36%)
12 Rise from floor	6 (46%)	3 (27%)	4 (36%)
13 Stand on heels	–	–	1 (9%)
14 Jump	1 (8%)	–	–
15/N16 Hop	–	–	–
17 Run	8 (62%)	5 (45%)	3 (27%)

Frequencies based on which item(s) participants deemed ‘most important’ on the NSAA. In some cases, participants selected more than one item, and some participants were not asked the question. Dashes (−) indicate that no participants mentioned that particular item as ‘most important.’

*High level: “Just running around being a kid, it’s important to jump and run. I don’t think it’s required in his daily life but if he couldn’t do it, he would be annoyed.” (CA-CG-01).*

*Mid level: “[03 Stand up from chair] was probably more important when he was in primary school … his chairs have been adapted for him …So, I would say now, the age he is, it doesn’t seem to be that important now” (UK-CG-010).*

*Low level: “Running, no, no. I think he’s probably very content if he could just continue to walk”* (US-CG-072).

This finding was also consistent with the interviewed physiotherapist’s definition of meaningful change. While only one caregiver described a meaningful change in relation to the total score, the majority of participants discussed meaningful change at the item level focusing on items that showed decline and the overall impact. As scores decline, items that are considered ‘nice to have’ showed less importance than items directly related to ADLs: *“Anything to do with ambulation and his ability to be independent and hold yourself up is really important…I would not rate it as the top thing in the world to be able to hop on one leg or stand on one leg. If he’s able to stand, great. Being able to stand on one leg, he’s lucky.” (UK-CG-032)*.

Participants were also asked about which items were considered important if they declined in the future, relative to items that were considered less important if they declined (see Supplementary Table 3). In the high score category, individuals said that items 01 Stand, 02 Walk, 03 Stand up from chair, and 17 Run would be most important if they declined. In the mid-score category, participants said that items 02 Walk, 06/08 Climb box step, and 11 Gets to sitting would be most important if they declined. In the low-score category, participants endorsed 02 Walk, 01 Stand, and 03 Stand up from chair to be most important if they declined. In each ability category these items reflect which items are most meaningful to participants at their current stage of ambulation.

#### Maintenance of functional abilities

**Figure 2:**
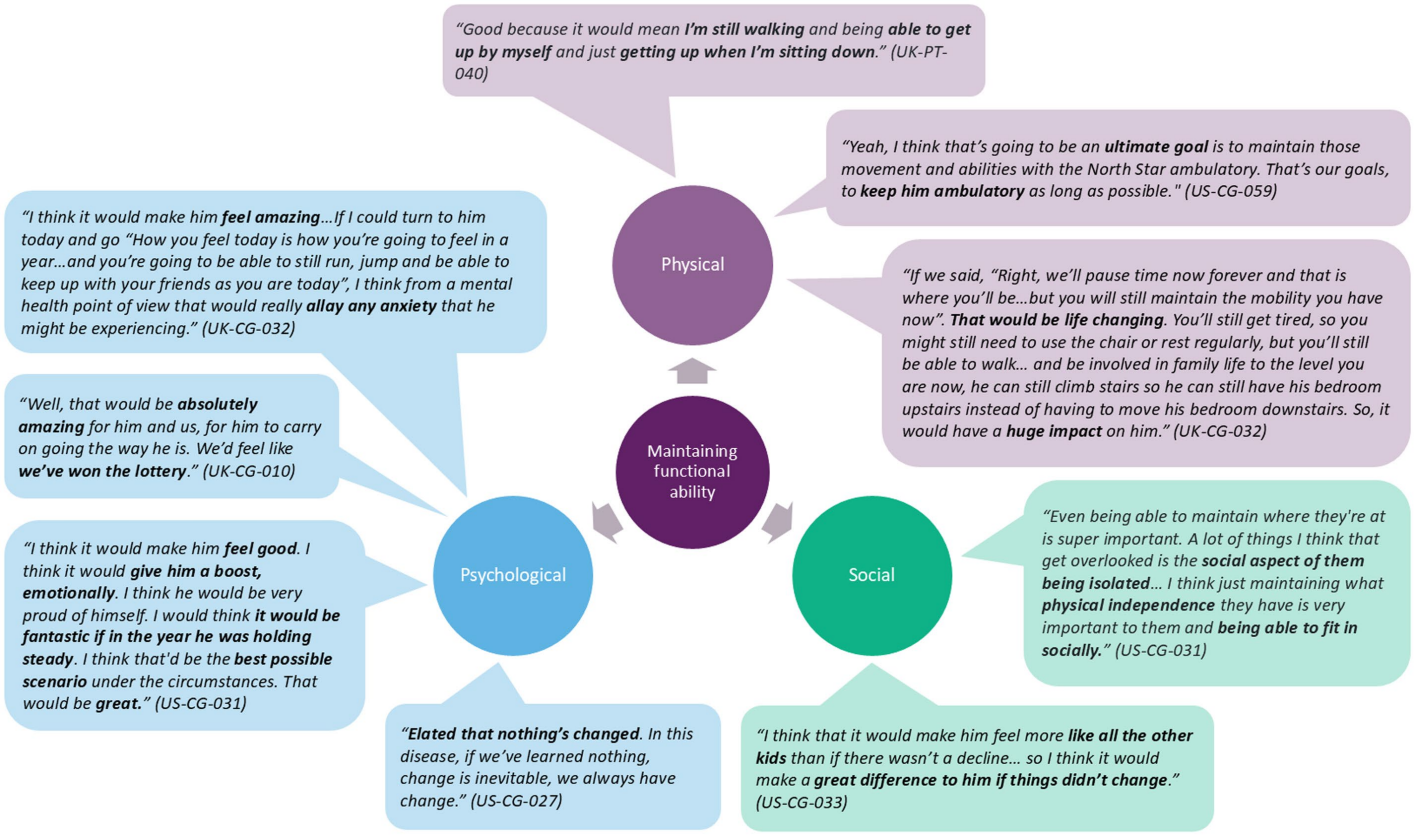
Impacts of maintenance associated with NSAA items.

Maintenance over one year was considered the ultimate goal. One caregiver said, *“if it improved he would absolutely love it and be very excited, but he’s content with his abilities … if that stays the same he’s absolutely fine,”* underscoring a common sentiment by participants. Figure 2 illustrates the varied impacts and positive aspects of maintaining current levels of functional ability. Walking was most important to maintain across ability scores, while other items differed in line with the trajectory of natural progression of DMD. Other items most important to maintain for high scorers were 17 Run, 02 Walk and 03 Stand up from chair; for mid-scorers, these were 02 Walk, 07/09 Descend box step, 01 Stand, and 17 Run; and for low-scorers, these were 02 Walk, 01 Stand, and 12 Rise from floor. See Supplementary Table 4 for individual items.

#### Full versus partial loss of function

Participants occasionally described impacts of a partial loss (score change 2 to 1) versus full loss (score change 1 to 0 or 2 to 0) of function on individual items. Apart from a shift from 2 to 0 – which was discussed less due to the progressive nature of decline – both full and partial loss correspond to a 1-point change, yet participants described differing impacts. In general, a full loss of function was reported as a more important decline than a partial loss, especially on items such as 03 Stand up from chair and 11 Gets to sitting, both of which were described by participants as having natural adaptations, meaning that a partial loss is easier to adapt to. This finding was observed generally across the sample rather than showing differences across ability categories. The one exception was for a caregiver whose son had a high NSAA score and was on an improving trajectory, who indicated that a decline from 2 to 1 on four items (stand, stand on one leg, step down, and run) would be important because he just wants to fit in and would get frustrated if he had to adapt to his current abilities. A full loss of 2-points (2 to 0) was described as being important by individuals in the high score category in a more hypothetical manner, for example, a caregiver said about 01 Stand, *“one day when he uses a chair, he’ll cope, but… for his lifestyle at the moment, it would be really disruptive if he could not do that anymore.” (UK-CG-037)*.

The impacts described between a partial and full loss differed in the intensity of impact. For example, a partial loss might result in some independence lost, some frustration, and a noticeable difference between the individual and their peers, whereas a full loss had associated impacts such as lost independence, being fully dependent on someone else, worsened mental health, and more falls and broken bones. On the difference between a partial and full-loss in 03 Stand up from chair, one caregiver said *“I do not think he would mind if it took him longer to get up… what would bother him more is if he was unable to get up without help because then he’d feel stuck” (US-CG-061).* Both clinicians described that a change from 2 to 1 might require changes to an individual’s daily life or more modifications, whereas a change from 1 to 0 would be more meaningful, “*they are in a 1 for long, long time and then from 1 to 0, it’s definitely a meaningful change”* (CL-1), and:

*“From 2 to 1, there is weakness going on, more significant weakness, and you will see some scores going probably down on the other items, but then from 1 to 0, yeah, it's a loss…”* (CL-1).

### PUL results

The key themes for ability scores in the PUL were related to independence and corresponded to dimensions of the PUL. The most important items were also related to functional ability and PUL dimension. Other key findings included (1) maintenance was considered important for all, with key items shifting across score groups; (2) small point changes had a big impact on independence; and (3) there were different impacts between partial and full loss in function.

#### Key themes by ability level

**Figure 3:**
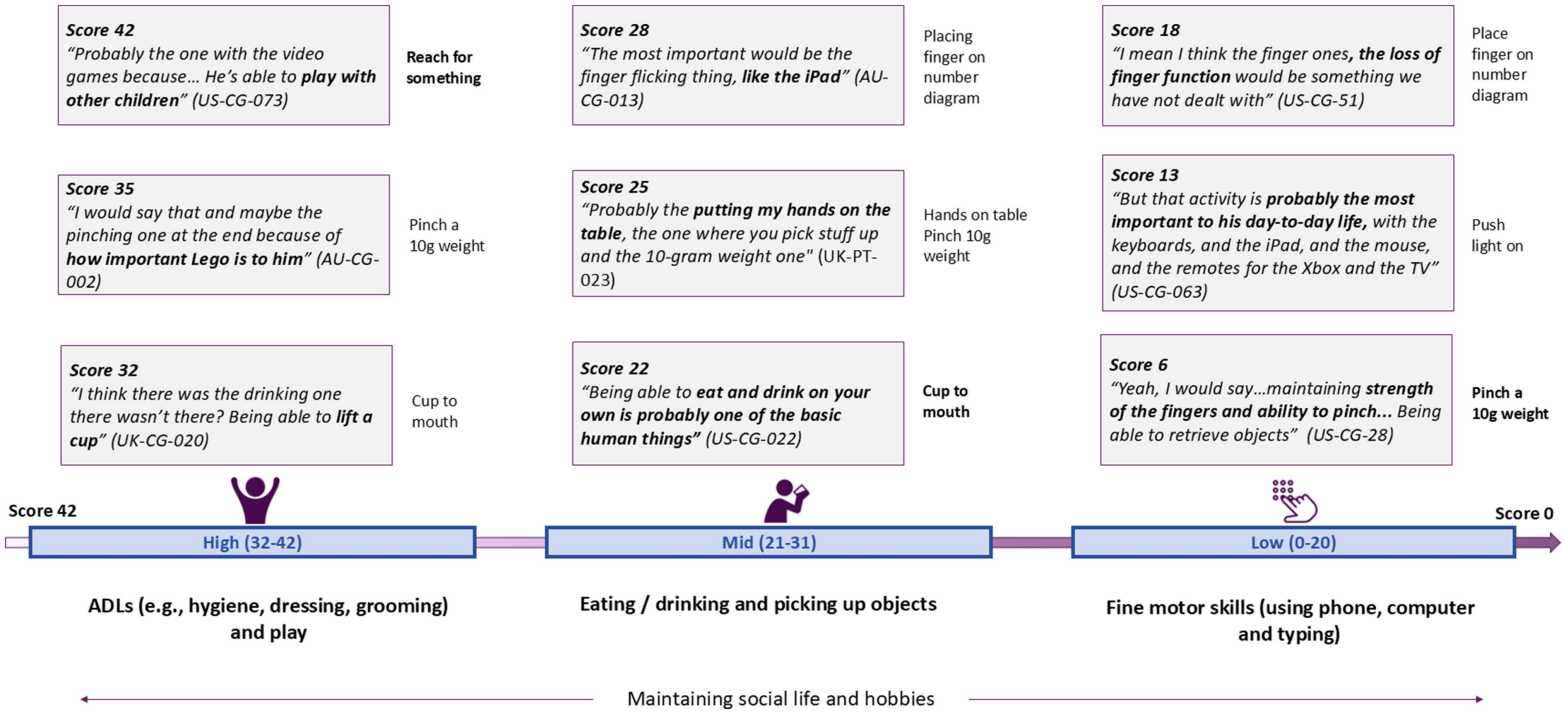
PUL ability scores and associated themes.

The items considered most important were related to functional ability and associated with PUL dimensions, as expected, e.g., the most important item to high-scorers was in the shoulder domain, to mid-scorers in the elbow-domain, and to low scorers in the distal domain (Table 5). Like the NSAA, a key theme for importance emerged from each ability score group (Figure 3).

**Table 5:** Most important items: frequencies for participants across PUL ability score categories high, mid, and low.

Item	High (32–42) (*n* = 14)	Mid (21–31) (*n* = 11)	Low (0–20) (*n* = 9)
01 Raise arms above head	2 (14%)	1 (9%)	–
02 Raise arms shoulder height	2 (14%)	–	–
03 Shoulder flexion to shoulder height	5 (36%)	3 (27%)	1 (11%)
04 Shoulder flexion to shoulder height 500 g	1 (7%)	–	–
05 Shoulder flexion above shoulder height 500 g	1 (7%)	–	–
06 Shoulder flexion above shoulder height 1 kg	–	1 (9%)	–
07 Hand(s) to mouth	5 (36%)	9 (82%)	3 (33%)
08 Hands to table from lap	2 (14%)	2 (18%)	1 (11%)
09 Move weight on table 100 g	2 (14%)	1 (9%)	2 (22%)
10 Move weight on table 500 g	1 (7%)	–	1 (11%)
11 Move weight on table 1 kg	–	–	–
12 Lift heavy can diagonally	–	1 (9%)	2 (22%)
13/14 Stack 3/5 cans	1 (7%)	–	1 (11%)
15 Remove lid from container	2 (14%)	–	–
16 Tearing paper	–	–	–
17 Tracing path	2 (14%)	–	4 (44%)
18 Push on light	3 (21%)	1 (9%)	3 (33%)
19 Supination	–	1 (9%)	–
20 Picking up coins	–	–	–
21 Placing finger on diagram	2 (14%)	1 (9%)	7 (78%)
22 Pick up 10 g weight	4 (29%)	1 (9%)	2 (22%)

Frequencies based on which item(s) participants deemed ‘most important’ on the PUL. In some cases, participants selected more than one item, and some participants were not asked the question. Dashes (−) indicate that no participants mentioned that particular item as ‘most important.’

Individuals with high PUL scores reported the importance of maintaining independence in ADLs, specifically related to grooming, personal hygiene (e.g., brushing teeth and showering), and dressing, all of which corresponded to items in the high-shoulder PUL domain. One of the most important items, 03 Reach in front, was discussed in relation to performing ADLs, but also for greeting others (e.g., with a fist-bump, hug, or handshake) and hobbies, including building Lego, playing videogames, and using a computer.

In the mid-score category participants discussed retaining independence in eating, drinking, and reaching. Picking up cutlery, receiving food, putting their hands on the table, or reaching out to grab a book, were all considered key. The most important item was 07 Bring cup to mouth, related to independent eating and drinking, and brushing teeth.

Participants with low scores discussed the importance of retaining independence in fine motor skills due to the need to communicate, control powerchairs, complete schoolwork, and maintain employment. In relation to the distal items declining, one caregiver said *“being a student is his identity right now. So, not being able to write or hold a pen like that would be pretty bad for him.” (US-PUL-063)*. For those with low scores on the PUL, the most important item was 21 Touch number on diagram, considered vital for using technology including mobile phones, iPads, remotes, and game controllers for communication.

#### Maintenance of functional abilities

Maintenance on the PUL was considered the ultimate goal, with key items corresponding to PUL ability level themes as described above (See Supplementary Table 5). A consistent theme across all participants interviewed on the PUL was that maintenance was important to continue hobbies and social lives, although the specific tasks and aspects of socialization differed across scores and age groups. Maintaining upper limb function was described as necessary for independence, and to “*do things by myself” (US-PT-002)*, as well as its importance above a decline:

*“I think in the day-to-day with Duchenne, it is declining. That’s the direction is going. It’s maintaining or declining. Maintaining is great, yeah. It’s a whole new mindset, but it feels as though you’re always marking a decline really.” (UK-CG-022)*

**Figure 4:**
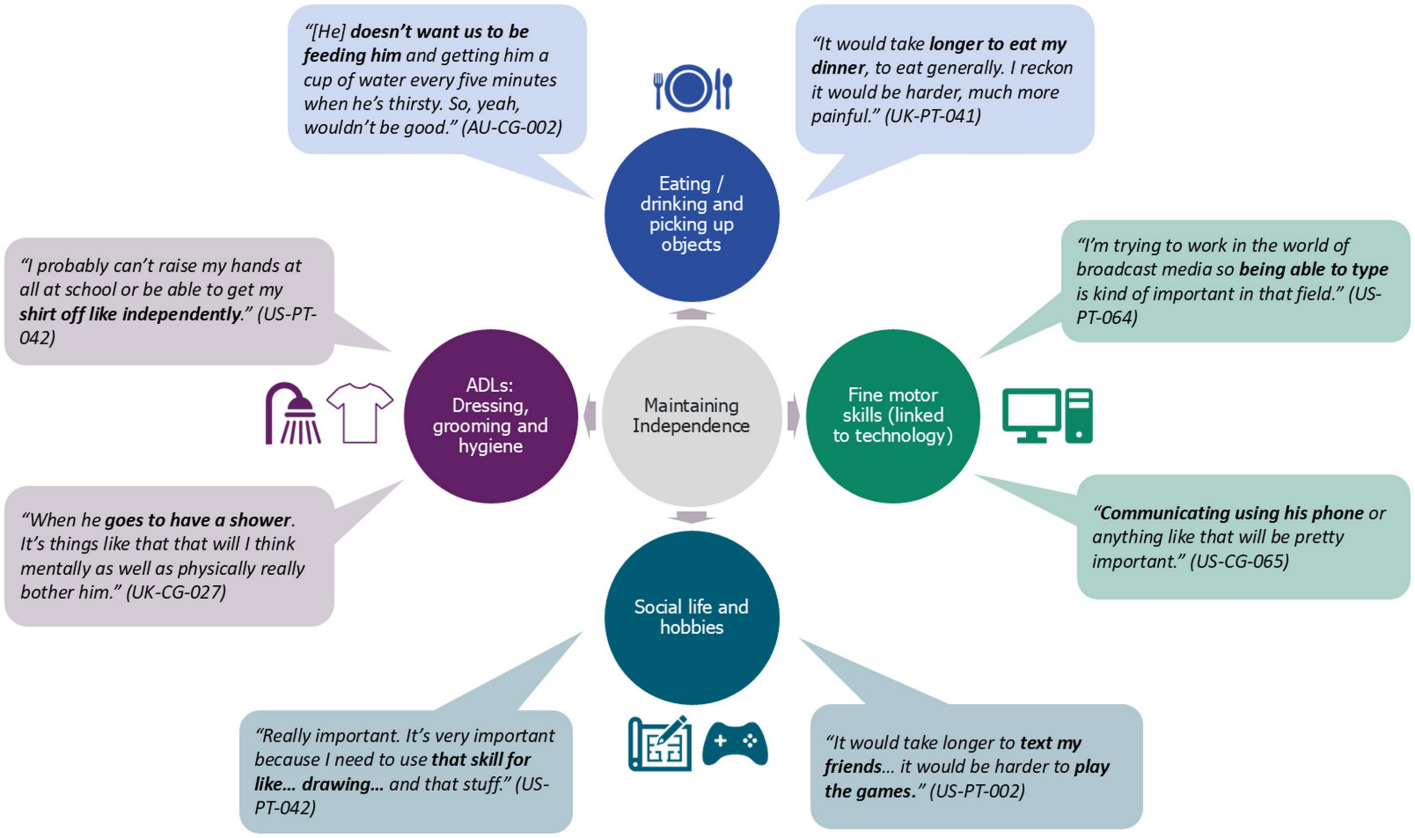
Impacts of maintenance associated with PUL items.

Figure 4 displays associated quotes from individuals with DMD and caregivers about the impacts associated with maintaining function across different themes.

#### Small point changes have a big impact on independence

The items that participants said they would most like to improve on were those linked to regaining abilities in independence-related tasks, e.g., getting dressed, reaching for objects, and personal hygiene (rather than points). Mid and low scorers consistently reported item 01 Raise arms above head as the function they would most like to improve. This item was not considered the most important in the shoulder domain for high scorers but was considered to increase independence and reduce reliance on a caregiver:

*“Being able to get dressed independently too. That's kind of a big thing as far as independence…It would just make things easier for me as far as not needing as much assistance. Yeah, not relying on a caregiver as much.” (US-PT-008)*

Some items were described in relation to a point improvement from 1 to 2. When discussing an improvement in relation to a change from 1 to 2 on 07 Cup to mouth, a caregiver said, *“It would be a huge, positive, significant life changing impact for him” (US-CG-065),* because being able to eat and drink by oneself has a huge impact on independence. The other items where a change from score 1 to 2 was specifically highlighted and deemed important were 22 Pinch 10 g weight, which if it got better *“would have an impact on his quality of life… to the extent that particularly for boys, that like playing games, video games, and remote controls require strength in your fingers” (US-CG-028),* and 05 Pass 500 g object.

When discussing meaningful change, the two physiotherapists said that the number of points considered meaningful differed depending on the participant’s PUL score. For example, a participant with a lower PUL score might find a loss of three points more meaningful than someone with a higher score. While caregivers and individuals with DMD did not necessarily talk about point-loss in the same way that clinicians did, they did note that change in DMD is gradual, and because adaptions are made over time, the number of points is not always the most important aspect of change:

*“He has been living with this for 15 years now, so he has slowly lost some of the things that he has been able to do. But since it’s a gradual thing… I think he is happy with what he is able to do…even when he lost the… ability to walk… he was escaping from the pain in his legs he was okay. He was accepting that change.” (US-PUL-069)*

#### Partial versus full loss of function

Losing function completely was considered more important on items related to independence and more meaningful than partial loss. One caregiver commented that a recent decline on 07 Cup to mouth from 2 to 1, “*does not feel that important because we have just changed what we do,”* however, “*not being able to drink yourself would be really important*” *(UK-CG-034)*.

When discussing a full loss of function (1 to 0), three further items emerged as key indicators of independence: 09 Move 100 g object on table, in relation to having to adapt surroundings, and 08 Move hand to table and 19 Pick up object and turn over, in relation to requiring more help from the caregiver:

*“I think that he’s at a point where he requires caregiver assistance for all these things. The caregiver’s just going to be doing 100% of that and his loss of independence to think that he can brush his teeth okay by himself or somebody has to brush his teeth for him. It’s another loss of an independent… that’s basic care.” (US-CG-051)*

Finally, the item that would be most important if it declined (in general) for all participants was 21 Placing finger on number diagram, given its relation to technology use. For the high-scorers and mid-scorers the next most important item was 03 Reach out. For the low-scorers only two other items were mentioned once each: 07 Hand to mouth and 19 Supination.

## Discussion

The objective of this qualitative study was to establish estimates of meaningful change from the perspective of individuals with DMD and their caregivers. Upon initial analysis, splitting the results into ability levels for each instrument made it possible to highlight key themes in each group to assist in summarizing disease progression. Subsequent analyses demonstrated that the most important or meaningful item(s) for individuals within these groups were relative to functional ability.

For the NSAA, key themes were related to ‘maximizing ability’, ‘modifying ability’ and ‘retaining independence’ as individuals progress from high to mid to low scores, respectively. The key themes in the PUL were linked by independence – with specific aspects differing based on ability score and functional ability – which in turn could be mapped on the PUL domains demonstrating that specific items’ meaningfulness shifts across the score range.

The second key finding for both the NSAA and PUL was the importance of maintenance of function, which should not be understated in a context where decline is inevitable. This finding was consistent across ability categories, with items that were considered most important to maintain also corresponding to functional ability. This supports the necessity of considering ≥0 points change on NSAA and PUL as a meaningful outcome for individuals with DMD.

Next, from a qualitative perspective on both instruments, meaningful change was described in relation to ability level per item, not necessarily change in total score or number of points, and different impacts were described between a full-loss and partial-loss of function. The findings diverged in the specificity of discussing point-changes between the NSAA and the PUL, likely because PUL changes are deemed as more gradual than NSAA, and more noticeable to clinicians than caregivers and individuals with DMD. In the NSAA interviews, individuals with DMD, their caregivers, and both clinicians described a partial loss holding less importance than a full loss. There was some evidence that a 1-point change can mean different things to participants depending on their ability score, which should be explored further in future studies.

Although point-change was not examined qualitatively from a total score level, participants across the study consistently reported how a 1-point decline relating to a lost function in relation to items connected to functional ability would be considered important to them. In the PUL, clinicians also highlighted that a partial loss was less important than a full loss across items, because while a compensation allows the activity to still be completed, a full loss means being unable to perform the task independently. Similarly, for caregivers and individuals with DMD, a full loss of function was considered more important on items that were directly related to independence, such as PUL item 07 Hands to mouth. The clinician interviews underscored the importance of considering baseline scores when interpreting meaningful change, especially on the PUL where losing a smaller number of points will have more impact with less mobility.

Finally, participants described how a small improvement (e.g., 1-point) on the PUL can translate to large impacts on independence. The items that were consistently described as being the most important to improve were those related to regaining independence in ADLs, such as being able to get dressed, reach for objects, and perform personal hygiene tasks.

### Interpretation

The rigor of these qualitative results is bolstered by the range of perspectives gathered and involvement of clinical experts, but also by similarities to the published lived experience of people with DMD. This research adds to the evidence base of recently published conceptual models (19–22) and qualitative reviews (23, 24) that include novel insights on concepts important to ambulatory and non-ambulatory individuals with DMD (19). In one study, which examined DMD qualitatively from the parent/patient perspective, the majority of ambulatory dyads interviewed indicated they would be satisfied with a treatment that stabilized disease progression (19), similar to the results of this study. Patient perspectives have also indicated treatment benefits differ based on disease progression. Younger children prioritize maintaining or improving mobility in muscle function compared to older children and adults who prioritize maintaining or improving independence and ADLs (25), supportive of the themes identified in NSAA and PUL in this study. Another recent study on meaningful change in Spinal Muscular Atrophy (SMA) (26), a hereditary motor neuron disease that also affects the muscles in a progressive manner, described similar results that stabilizing the disease and preventing further loss would be seen as a huge benefit.

While this study largely examined qualitative perspectives and did not ask about meaningful point-changes at a total score level, the findings clearly indicate 0-point changes (maintenance) are considered important to individuals with DMD on both the NSAA and the PUL, as well as single point changes, although the specific items vary based on their relative disease-stage. This was especially evident in the PUL results, where small-point changes (e.g., 1-point improvement) would incur important knock-on effects to being able to conduct ADLs independently.

Preventing the loss in one activity on the NSAA and deterioration in at least two activities has been hypothesized to be meaningful to parents/patients (27). This study has added to the literature indicating that there is a clinical difference between a partial and full loss of function, first described by McDonald et al. (28) and expanded upon in a recent paper (16). Our findings from the NSAA further bolster this hypothesis by describing the different impacts between the effect of a 1-point decline or improvement on individual items. The insight from clinicians also indicated that this was the case in the PUL, although individuals with DMD and caregivers tended to discuss these differences less. Clinicians also highlighted the importance of the percentage of completely lost functions versus being able to accomplish items with compensation; although this was not fully documented in this study, it is an area that requires future research. These results, along with recently published (29) data on NSAA total scores in the context of stability or decline, can benefit families and clinicians in treatment management.

In summary, these findings underscore the necessity of considering ability as part of the context for interpreting meaningful change and support the inference that more granular results are needed to understand what is most meaningful to patients at specific stages of disease progression. The total score and functional ability of the individual should be taken into consideration when interpreting trial results. This approach of a combined, comprehensive metric might aid the interpretability of published MCID estimates (16) from quantitative data by leveraging which items are most meaningful at different ability levels across disease progression.

### Limitations

It is important to acknowledge some limitations in interpreting the study findings. While best efforts were made to recruit as wide a sample as possible by setting quotas it is likely some selection bias occurred due to those active within PAGs being more likely to volunteer. Recruitment in rare disease is challenging, and substantial barriers exist recruiting pediatric participants with neuromuscular disease, such as DMD (30). The recruitment approach we took resulted in a less diverse sample than originally anticipated, with participants skewed toward a white and educated population. The sample also deviated in some respects from the original quota targets due to the relevance of instruments in patient populations after LOA, making it difficult to recruit more individuals with DMD in the NSAA sample and skewing the results toward caregivers. The study was confined to English-speaking countries, with the majority of the sample recruited from the UK and US, and only four participants interviewed from Australia and two participants from Canada. Furthermore, three physiotherapists were initially recruited for the study, but one individual dropped out prior to the interview. Additional insights from clinicians in the other countries investigated would have strengthened the results of the study even further. Future work should explore qualitative insights on the NSAA and PUL from a more diverse sample, more individuals with DMD capturing the perspective of the NSAA, more countries, and non-English speaking populations.

No saturation analysis was conducted which could be considered another limitation. However, as saturation is typically reached after the first twelve qualitative interviews, the sample size for each instrument was generally deemed sufficient to surpass any new themes arising (28, 31). Further, given the intent was to understand meaningful change and not develop a new patient-reported outcome measure, traditional saturation analysis as typically required for concept elicitation interviews was deemed unnecessary in this context (32).

A further limitation should be admitted in the study design itself. The interview guides were drafted to ensure their acceptability to participants of all ages, meaning certain questions were removed that explicitly asked about point-changes. Next, because of the sensitivity of the interviews, not all individuals were asked every question; we chose a semi-structured approach to balance obtaining in-depth meaningful data with the sensitivity required when engaging with participants, some who were young, living with a degenerative disease such as DMD. Individuals who responded to all interview parts were thus more likely to be those with higher scores or who were more able. This increased the number of responses in the high-score category for items considered ‘most important,’ which could indicate an implicit bias in interpreting these results.

In terms of the qualitative analysis, the mean ages within the NSAA ability groups were quite similar and could have captured a wider DMD population. The groups were defined prior to conducting the analysis, however, which removed any bias from comparing results between groups. Next, there were four participants in the NSAA sample (between age 4–6) who were likely on an improving trajectory versus individuals who were mainly declining. This was not factored into the coding guide or inclusion criteria and therefore all participants were analyzed according to ability categories separate to whether they might be improving or declining.

Finally, there were some small discrepancies between the clinician-reported scores and patient-reported scores, meaning some participants fell into two ability categories. The clinician-reported scores were selected to form these groups given their objectivity, even though the patient-reported scores guided the interviews.

### Directions for future work

Additional qualitative research with individuals with DMD is warranted, especially with younger individuals. The boys interviewed provided rich insights into their own experiences. A future consideration would be to separate the sample of younger individuals based on whether they were on an improving or declining trajectory. Finally, this research provides an opportunity to support existing quantitative estimates and produce a more robust MCID that considers the totality of evidence available.

## Conclusion

This study emphasized how the perception of meaningful change is influenced by ability levels and ambulatory function, with participants describing their need to maximize certain abilities, maintain function, and retain independence depending on their NSAA score. For the PUL, participants underscored the importance of maintenance of their functional abilities, and highlighted key themes related to maintaining independence in ADLs, reaching out and eating/drinking, and finger function for technology use across score categories.

Understanding the perspective of individuals with DMD and caregivers of what is considered meaningful across ability scores on the NSAA and PUL adds important context when interpreting improvement, decline and maintenance of functional ability. The results for both instruments highlighted differences between partial and full loss of function, the relationship between meaningful change and functional ability, and the importance of maintenance as a treatment benefit, whilst also showing which items are considered most important to individuals to lose at different stages of disease severity.

In summary, this study was an ambitious undertaking and, to our knowledge at time of writing, the largest qualitative study to date focused on defining what constitutes a meaningful change on the NSAA and PUL from the perspective of individuals with DMD and their caregivers. The results provide context on how to interpret changes in NSAA and PUL scores in DMD clinical trials, offering an important contribution to the literature and paving the way for future work in qualitative meaningful change.

## Data Availability

The datasets presented in this article are not readily available because they are qualitative transcripts and cannot be completely deidentified. Requests to access the datasets should be directed to aviva.gillman@modusoutcomes.com.
