# Supplementary material for: A patient-centered qualitative evaluation of meaningful change on the NSAA and PUL in Duchenne Muscular Dystrophy
Source: Front Neurol. 2025 Mar 4;16:1509174. doi: 10.3389/fneur.2025.1509174 (PMC11915531; doi:10.3389/fneur.2025.1509174)
Supplement: Supplementary file 2 [file Table_2.docx]

*Table S2 Participants eligibility into the study by country*

|  | ***Australia (n)*** | ***Canada (n)*** | ***UK (n)*** | ***US (n)*** |
| --- | --- | --- | --- | --- |
| Contacted interviewer team | 12 | 6 | 41 | 74 |
| Returned screener | 4 | 3 | 26 | 50 |
| Eligible | 4 | 2 | 22 | 44 |
| Ineligible | 0 | 1 | 4 | 16 |
| Did not return screener | 6 | 3 | 15 | 14 |
| No longer interested | 2 | 3 | 0 | 4^†^ |
| Participated | 4 | 2 | 23* | 40 |

Table shows the number of participants who reached out to the interview team as a result of the PAG email blasts, across each country, as well as those who returned or did not return the screener, those who were eligible or not eligible, those who dropped out, and those who participated.

*One participant (individual with DMD) in the UK was interviewed twice because he completed the NSAA and PUL and was on the cusp of both

†4 participants in this category had already consented; participants in other countries in this category were not consented
