# Supplementary material for: A patient-centered qualitative evaluation of meaningful change on the NSAA and PUL in Duchenne Muscular Dystrophy
Source: Front Neurol. 2025 Mar 4;16:1509174. doi: 10.3389/fneur.2025.1509174 (PMC11915531; doi:10.3389/fneur.2025.1509174)
Supplement: Supplementary file 3 [file Table_3.docx]

*Table S3 Importance of decline: frequencies for participants across NSAA ability score categories high, mid, and low*

| *Item* | **High (25-34)**  **(n=13)** | | **Mid (15-24)**  **(n=11)** | | **Low (0-14)**  **(n=11)** | |
| --- | --- | --- | --- | --- | --- | --- |
|  | **Important decline** | **Less important decline** | **Important decline** | **Less important decline** | **Important decline** | **Less important decline** |
| 01 Stand | 8 (62%) | 3 (23%) | 4 (36%) | 5 (45%) | 6 (55%) | 0 (0%) |
| 02 Walk | 8 (62%) | 0 (0%) | 7 (64%) | 0 (0%) | 7 (64%) | 2 (18%) |
| 03 Stand up from chair | 7 (54%) | 4 (31%) | 6 (55%) | 6 (55%) | 6 (55%) | 0 (0%) |
| 04/05 Stand on one leg | 3 (23%) | 6 (46%) | 2 (18%) | 3 (27%) | 0 (0%) | 2 (18%) |
| 06/08 Climb box step - right and left | 5 (38%) | 1 (8%) | 7 (64%) | 0 (0%) | 1 (9%) | 1 (9%) |
| 07/09 Descend box step - right and left | 6 (46%) | 0 (0%) | 4 (36%) | 0 (0%) | 2 (18%) | 1 (9%) |
| 10 Lifts head | 2 (15%) | 6 (46%) | 4 (36%) | 2 (18%) | 4 (36%) | 1 (9%) |
| 11 Gets to sitting | 6 (46%) | 4 (31%) | 8 (73%) | 2 (18%) | 4 (36%) | 1 (9%) |
| 12 Rise from floor | 6 (46%) | 2 (15%) | 3 (27%) | 0 (0%) | 2 (18%) | 0 (0%) |
| 13 Stand on heels | 0 (0%) | 8 (62%) | 0 (0%) | 4 (36%) | 1 (9%) | 2 (18%) |
| 14 Jump | 4 (31%) | 3 (23%) | 0 (0%) | 5 (45%) | 1 (9%) | 1 (9%) |
| 15/N16 Hop - right and left | 1 (8%) | 5 (38%) | 0 (0%) | 2 (18%) | 0 (0%) | 0 (0%) |
| 17 Run | 7 (54%) | 1 (8%) | 4 (36%) | 2 (18%) | 4 (36%) | 1 (9%) |

Table shows item participants reported would be ‘important’ vs ‘less important’ if they declined. Not all participants answered whether an item would be important or less important if it declined; frequencies therefore do not add up to total transcript numbers in each category.
