# Supplementary material for: A patient-centered qualitative evaluation of meaningful change on the NSAA and PUL in Duchenne Muscular Dystrophy
Source: Front Neurol. 2025 Mar 4;16:1509174. doi: 10.3389/fneur.2025.1509174 (PMC11915531; doi:10.3389/fneur.2025.1509174)
Supplement: Supplementary file 4 [file Table_4.docx]

*Table S4 Importance of maintenance: frequencies for participants across NSAA ability score categories high, mid, and low*

| ***Item*** | ***High score (25-34) (n=13)*** | ***Mid score (15-24) (n=11)*** | ***Low score (0-14) (n=11)*** |
| --- | --- | --- | --- |
| 01 Stand | 2 (15%) | 3 (27%) | 2 (18%) |
| 02 Walk | 6 (46%) | 6 (55%) | 3 (27%) |
| 03 Stand up from chair | 5 (38%) | 1 (9%) | - |
| 04/05 Stand on one leg | - | - | - |
| 06/08 Climb box step | - | 2 (18%) | - |
| 07/09 Descend box step | 1 (8%) | 3 (27%) | - |
| 10 Lifts head | 1 (8%) | 2 (18%) | - |
| 11 Gets to sitting | - | 1 (9%) | - |
| 12 Rise from floor | 3 (23%) | 2 (18%) | 1 (9%) |
| 13 Stand on heels | - | - | - |
| 14 Jump | - | - | - |
| 15/N16 Hop | - | - | - |
| 17 Run | 7 (54%) | 3 (27%) | - |

Items participants reported they would ‘most like to maintain’ on the NSAA. Not all participants answered whether an item would be important to maintain; frequencies therefore do not add up to total transcript numbers in each category
