# Supplementary material for: A patient-centered qualitative evaluation of meaningful change on the NSAA and PUL in Duchenne Muscular Dystrophy
Source: Front Neurol. 2025 Mar 4;16:1509174. doi: 10.3389/fneur.2025.1509174 (PMC11915531; doi:10.3389/fneur.2025.1509174)
Supplement: Supplementary file 5 [file Table_5.docx]

*Table S5 Importance of maintenance: frequencies for participants across PUL ability score categories high, mid, and low*

| ***Item*** | ***High score (32-42) (n=14)*** | ***Mid score (21-31) (n=11)*** | ***Low score (0-20) (n=9)*** |
| --- | --- | --- | --- |
| 01 Raise arms above head | 1 (7%) | - | - |
| 02 Raise arms shoulder height | - | 1 (9%) | - |
| 03 Shoulder flexion to shoulder height | 2 (14%) | 1 (9%) | - |
| 04 Shoulder flexion to shoulder height 500g | 1 (7%) | 1 (9%) | - |
| 05 Shoulder flexion above shoulder height 500g | 1 (7%) | 2 (18%) | - |
| 06 Shoulder flexion above shoulder height 1kg | - | 1 (9%) | - |
| 07 Hand(s) to mouth | 5 (36%) | 6 (55%) | 3 (33%) |
| 08 Hands to table from lap | - | 1 (9%) | 2 (22%) |
| 09 Move weight on table 100g | - | 1 (9%) | 2 (22%) |
| 10 Move weight on table 500g | - | - | - |
| 11 Move weight on table 1kg | - | - | - |
| 12 Lift heavy can diagonally | - | 1 (9%) | 1 (11%) |
| 13/14 Stack 3/5 cans | - | 2 (18%) | - |
| 15 Remove lid from container | - | 1 (9%) | - |
| 16 Tearing paper | - | - | - |
| 17 Tracing path | 1 (7%) | - | 2 (22%) |
| 18 Push on light | 2 (14%) | - | 1 (11%) |
| 19 Supination | - | - | - |
| 20 Picking up coins | - | - | - |
| 21 Placing finger on diagram | 1 (7%) | 4 (36%) | 1 (11%) |
| 22 Pick up 10g weight | 2 (14%) | - | 2 (22%) |

Items participants reported they would ‘most like to maintain’ on the PUL. Not all participants answered whether an item would be important to maintain; frequencies therefore do not add up to total transcript numbers in each category.
